# Regulation and bioinformatic analysis of circ_0015891/miR-129-1-3p axis in methamphetamine-induced dopaminergic apoptosis

**DOI:** 10.3389/fendo.2022.999211

**Published:** 2022-09-20

**Authors:** Bingpeng Deng, Xuan Tang, Yong Wang

**Affiliations:** Department of Forensic Science, School of Basic Medical Science, Central South University, Changsha, China

**Keywords:** miR-129-1-3p, apoptosis, circ_0015891, methamphetamine (METH), bioinformatic analysis

## Abstract

Methamphetamine (METH) abuse can result in severe neurotoxicity, for which the mechanism is not yet clear. In the present study, we investigated the role of noncoding RNAs in METH-induced dopaminergic neurotoxicity, and analyzed the underlying mechanism using bioinformatic methods. We confirmed by flow cytometry that miR-129-1-3p is involved in promoting dopaminergic apoptosis under METH treatment and its role could be inhibited by a high concentration of circ_0015891. Also, we combined transcriptomic data with bioinformatics to explore the downstream mechanism of miR-129-1-3p regulation of METH-induced apoptosis, highlighted the potentially pivotal figure of response to nutrition. Further bioinformatic analysis of circ_0015891 was conducted as well and showed that circ_0015891 was the sponge of various microRNAs that effect apoptosis by different mechanisms. Collectively, we found a novel circ_0015891/miR-129-1-3p axis that may be a promising therapeutic target for METH-induced dopaminergic neurotoxicity.

## Introduction

Methamphetamine (METH), a psychoactive drug derived from amphetamine, is globally and seriously abused on a large scale due to its high potential for addiction and relatively low difficulty to access (1, 2). In addition to its potent addictive properties, METH also causes neurotoxicity that cannot be ignored. More than 4 decades ago, METH-induced dopaminergic toxicity was reported (3, 4). Multiple mechanisms concerning apoptosis, the generation of reactive oxygen and nitrogen species, hyperthermia and aberrant dopamine/glutamate transmission were uncovered to explain METH-caused impairment to dopaminergic cells (5, 6). However, the underlying mechanisms involved in dopaminergic toxicity induced by METH are still not completely determined and need more exploration.

MicroRNAs (miRNAs/miRs) are a species of short, evolutionarily conserved, single-stranded linear non-coding RNAs with a length of 18-25 nucleotides (7). Numerous studies have confirmed that miRNAs play crucial roles in various pathophysiological processes that encompass psychoactive drug-related pathways through complementary base pairing with targeted mRNAs (8). A previous study verified that miR-143 reduced METH-mediated microglial apoptosis by targeting BBC3 (9), whereas studies focusing on the role of miRNAs in dopaminergic apoptosis are still lacked.

Circular RNAs (circRNAs) are a class of covalently closed loop noncoding RNAs that are more stable than their associated linear RNA (10, 11). As an endogenous sponge of targeted miRNAs, circRNA functions in suppressing miRNAs. It has been proven that circHIPK2 binds and thus inhibits miR-124-2HG, resulting in the astrocyte activation under METH treatment (12). However, whether circRNA/miRNA axes are involved in mediating METH-induced dopaminergic apoptosis is mainly unknown.

The present study demonstrated that circ_0015891/miR-129-1-3p axis regulated METH-induced dopaminergic apoptosis, and analyzed underlying mechanism by using bioinformatic methods.

## Methods

### Cell culture

The human neuroblastoma cell line SH-SY5Y was purchased from Shanghai Zhongqiao Xinzhou Biotechnology Co., Ltd (Shanghai, China) and cultured in minimum essential medium (MEM, 45%; Shanghai Zhongqiao Xinzhou Biotechnology Co., Ltd) supplemented with 10% fetal bovine serum, 1% sodium pyruvate and 1% penicillin/streptomycin and F12 medium (45%). Incubator for cell culture was set to 37°C and 5% CO2.

### Extraction and sequencing of RNA

The METH group was treated with 2 mM METH for 24 hours and the control group was treated with the same volume of PBS for 24 hours. The cultured cells were counted and washed twice with PBS. The appropriate amount of TRIzol^®^ reagent (Sigma-Aldrich, Saint Louis, MO USA) was added to the lysate and repeatedly blown until complete lysis was achieved. The lysate (1.5 ml) was transferred to a centrifuge tube without enzyme and stored at -80°C. The integrity of RNA was assessed using agarose gel electrophoresis. The quality of RNA was checked using a NanoDrop spectrophotometer (Thermo Fisher Scientific, Waltham, MA, USA). Transcriptome sequencing was completed using the HiSeq^®^ sequencing platform (Illumina, San Diego, CA, USA), and differentially expressed genes (DEGs) were analyzed using DESeq2. Experiments were performed in triplicate for each experimental group.

### Reverse transcriptase quantitative polymerase chain reaction

Total RNA from SH-SY5Y cell line was extracted using TRIzol^®^ reagent (Thermo Fisher Scientific, Waltham, MA, USA) and absorbance values were measured at 260 nm vs. 280 nm using a UV spectrophotometer to calculate RNA concentration and purity. Depending on the amount of RNA used, the appropriate concentration of ATP was diluted. miRNA Reverse Transcription Kit (Beijing ComWin Biotech Co., Ltd, Beijing, China) was used to complete the reverse transcription of the extracted RNA in two steps: 1. Mixing total RNA, diluted ATP and E.coli Poly (A) polymerase to prepare Poly(A) reaction solution 2. The Poly(A) reaction solution was mixed with dNTPs, RT primer and other auxiliary reagents with an incubation for 50 min. The synthesized cDNA reaction solution can be directly used for fluorescence quantitation assay or stored at -20°C for backup. Quantitative PCR was using SYBR Green method. H-U6 was the internal reference gene for the miRNA assay with forward primers F (CTCGCTTCGGCAGCACA), reverse primers R (AACGCTTCACGAATTTGCGT). The sequences of the synthesized miRNAs are: miR-1233-3p (TGAGCCCTGTCCTCCCGCAG), miR-151a-3p (CTAGACTGAAGCTCCTTGAGG), miR-31-5p (AGGCAAGATGCTGGCATAGCT), miR-532-3p (CCTCCCACACCCAAGGCTTGCA), miR-1225-5p (GTGGGTACGGCCCAGTGGGG ), miR-615-5p (GGTCCCCGGTGCTCGGATC), miR-107 (AGCAGCATTGTACAGGGCTATCA), miR-3127-5p (ATCAGGGCTTGTGGAATGGGAAG), miR-193a-5p (TGGGTCTTTGCGGGCGAGATGA), miR-129-1-3p (AGCCCTTACCCCAAAAAGTATAA). H-GAPDH was the internal reference gene for the circRNA assay with forward primers F (ACAGCCTCAAGATCATCAGC), reverse primers R (GGTCATGAGTCCTTCCACGAT). Primers for circ_0015891 were F (TCCCTTTAACCCAAGACCCTGC) and R (CTTGCAGTAAATCTCCTCACCAT). All primers were synthesised by Sangon Biotech Co., Ltd (Shanghai, China).

### Flow cytometry detection of apoptosis

SH-SY5Y cells were transfected with circ_0015891 plasmid with a final system concentration of 1-4 μg/mL) or inhibitor/mimic of miR-129-1-3p using Lipofectamine™2000 (Invitrogen, Carlsbad, CA, USA). The METH-treatment groups were given a final METH concentration of 2 mM and the control groups were given PBS added to the same volume. The treated cells were incubated in an incubator at 37°C and 5% CO2 for 24 h. After EDTA-free trypsin digestion and three gentle washes of PBS, the treated cells were collected stained using the Annexin V-APC Apoptosis Kit (KeyGEN BioTECH, Jiangsu, China). 5-15min reaction at room temperature with no light and then apoptosis was determined by flow cytometry. Flowjo software was used for apoptosis analysis. The sequences of the miR-129-1-3p mimic-NC were sense (5’-UUCUCCGAACGUGUCACGUTT-3’) and antisense (5’-ACGUGACACGUUCGGAGAATT-3’). The sequences of the miR-129-1-3p inhibitor-NC was 5’-CAGUACUUUUGUGUAGUACAA-3’.

### Dual-luciferase reporter assay

The circ_0015891 dual luciferase reporter vector (wild type) pHG-MirTarget-circ_0015891 was purchased from HonorGene Co., Ltd (Changsha, China) and was transfected into cells with Lipofectamine™2000 (Invitrogen, Carlsbad, CA, USA). Dual-luciferase reporter assay kit (Promega, Madison, WI, USA) was used for activating luciferase activity, which was then detected by Chemiluminescence detector.

### Bioinformatic analysis and visualization

Gene Ontology (GO) analysis of mRNAs was conducted by online database David (https://david.ncifcrf.gov/home.jsp) (13). Metascape (https://metascape.org/) was utilized to enrich and analyze processes and pathways of mRNAs (14). Further analysis was performed using Cytoscape software. Pathway analysis of miRNAs was conducted by DIANA-miRPath tool (http://www.microrna.gr/miRPathv3) (15). Heatmap, volcano plot, string diagram and bi-directional bar graph were visualized by (https://www.bioinformatics.com.cn). ENCORI was applied to predict proteins targeted by miR-129-1-3p and miRNAs sponged by circ_0015891 (16). Binding sites between circ_0015891 and miRNA were calculated by miRanda (cbio.mskcc.org/miRNA2003/miranda.html) (17, 18). METH-related protein were collected by using OMIM (www.omim.org) (19), and GeneCards (https://www.genecards.org) (20), and DrugBank Online (https://go.drugbank.com) (21).

### Statistical analysis

Experiments were carried out at least thrice. Comparisons between the two groups were determined using two-tailed unpaired the student’s t test. All data except for bioinformatic data were analyzed using GraphPad Prism software. Significance was set at *p < 0.05.

## Results

### Identification of miR-129-1-3p from the DEGs between METH-treated SH-SY5Y cells and control group

**Figure 1:**
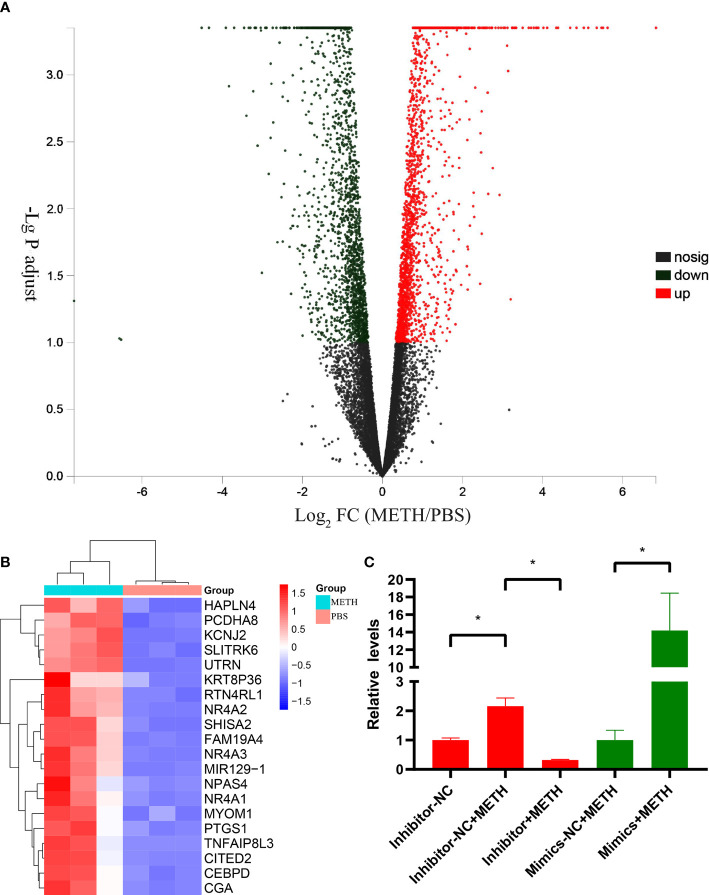
DEGs between METH-treatment group and PBS-treatment group in SH-SY5Y cell lines. **(A)** Volcano plots of DEGs. The horizontal coordinates are the values of the fold change in gene expression differences and the vertical coordinates are the values of the statistical tests for differences in gene expression changes, both logarithmically processed. Each point in the graph represents a specific gene, red points indicate significantly up-regulated genes, blue points indicate significantly down-regulated genes, and black points are insignificantly differentially expressed genes. **(B)** Heatmap of top 20 up-regulated DEGs. Three samples were tested in each of the METH and PBS groups, and the relative expression of genes is indicated by red or blue squares. **(C)** Relative expression levels of miR-129-1-3p tested by RT-qPCR. Significant differences are indicated by asterisks. Error bars represent the mean ± SEM. *P<0.05.

As shown in our previous transcriptome sequencing results (22), plenty of genes expressed differentially with a significance of adjusted P value < 0.1 in METH-treatment group versus PBS-treatment group in SH-SY5Y cell lines: 2055 genes were up-regulated, 2046 genes were down-regulated. The distribution of the gene expression is visualized in the corresponding volcano plot (**Figure 1A**). The top-20 up-regulated genes under METH treatment were shown in a heatmap (**Figure 1B**). Most of these genes participate in regulation of apoptosis in different types of cells including neurons. As the only non-coding RNA in the top 20 up-regulated genes, miR-129-1-3p was ranking 12^th^ (**Figure 1B**). The up-regulated expression of miR-129-1-3p under METH treatment was subsequently validated by RT-qPCR experiment (**Figure 1C**).

### MiR-129-1-3p mediates METH-induced dopaminergic apoptosis

**Figure 2:**
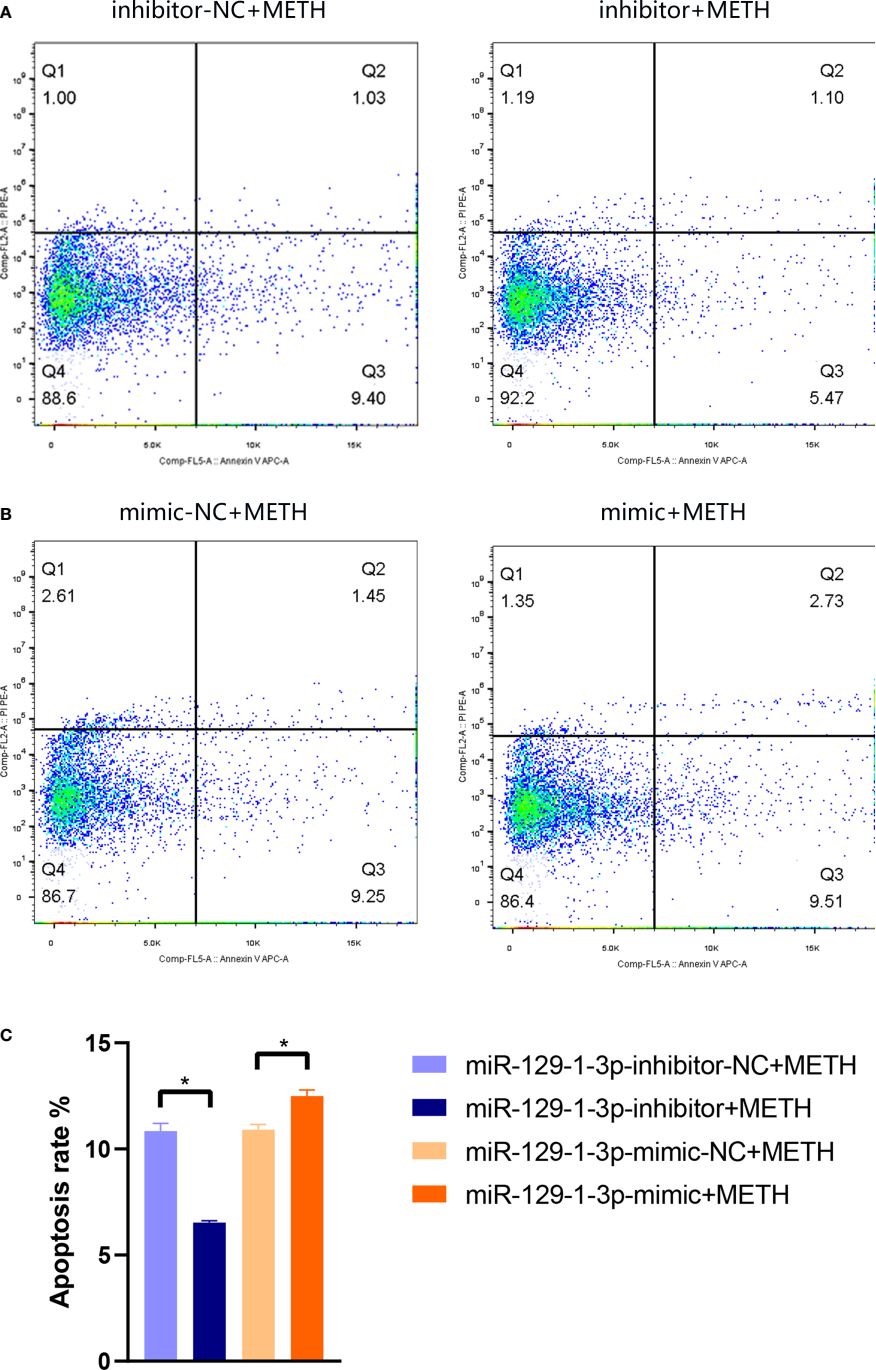
Flow cytometry analysis of apoptotic SH-SY5Y cells in **(A)** miR-129-1-3p inhibitor-NC + METH group and miR-129-1-3p inhibitor + METH group and **(B)** miR-129-1-3p mimic-NC group + METH and miR-129-1-3p mimic group + METH. **(C)** The apoptosis rate = percentage of early apoptosis (Q3) + percentage of late apoptosis (Q2). Significant differences are indicated by asterisks. Error bars represent the mean ± SEM. *P<0.05. mimic-NC: miR-129-1-3p mimic-NC, inhibitor-NC: miR-129-1-3p inhibitor-NC.

To investigate the role of miR-129-1-3p in neurotoxicity induced by METH, flow cytometry was conducted to detect apoptosis rate of SH-SY5Y cell line. The apoptosis rate was 10.84 ± 0.36% and 6.53 ± 0.09% when treated with miR-129-1-3p inhibitor-NC + METH and miR-129-1-3p inhibitor + METH, respectively (**Figure 2A**). The apoptosis rate was 10.90 ± 0.26% and 12.50 ± 0.28% when treated with miR-129-1-3p mimic-NC + METH and miR-129-1-3p mimic + METH (**Figure 2B**). Compared to the control group, miR-129-1-3p inhibitor effectively reduced METH-induced apoptosis rate especially early apoptosis (Q3) rate which was somewhat increased by miR-129-1-3p mimic (**Figure 2C**). These results support the role of miR-129-1-3p in promoting METH-induced apoptosis in dopaminergic cells.

### Bioinformatic analysis indicates the involved pathways and processes and potential targets of miR-129-1-3p

**Figure 3:**
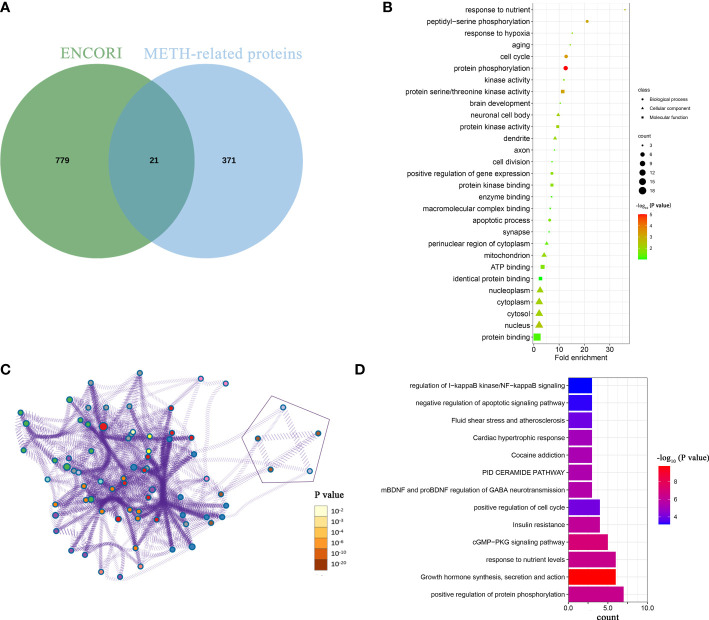
Bioinformatic analysis of downstream mechanism of miR-129-1-3p in METH-induced dopaminergic apoptosis. **(A)** Venn intersection of possible targets of miR-129-1-3p from ENCORI and METH-related proteins. **(B)** GO analysis of 21 proteins. This panel shows the top 10 enriched items for each of the biological process and molecular function and cellular component. Fold enrichment = (enriched gene counts/pop hits)/(list total gene counts/pop total hits). **(C)** pathway and process analysis of 21 proteins by Metascape. Each process or pathway was represented by a single dot. The line between the dots means that there is an overlap between the proteins enriched by the two processes or pathways. The 5 points representing apoptosis-related processes or pathways were enclosed by geometric figures. **(D)** Enriched pathways and processes of 21 proteins sorted by enriched gene counts from Metascape.

To explore the downstream mechanism of miR-129-1-3p in METH-induced neurotoxicity, bioinformatic analysis was conducted. Possible targets of miR-129-1-3p were predicted by ENCORI (16). Proteins related to METH responses were obtained from 3 online databases: GeneCards, OMIM and DrugBank Online (19–21). The Venn intersection showed that 21 METH-related proteins were potential targets of miR-129-1-3p (**Figure 3A**).

GO analysis of 21 proteins displayed that response to nutrient, peptidyl-serine phosphorylation, response to hypoxia, aging and cell cycle were top-5 enriched biological processes sorted by fold enrichment (**Figure 3B**) (13). The apoptotic process was ranking the 10^th^ in biological process, which contains 4 genes: MAP2K4, PRKCE, PAWR, and MAPK3. These genes may be targeted by miR-129-1-3p and thus promote METH-induced dopaminergic apoptosis.

In order to not miss genes that may function in apoptosis, Metascape, a tool that integrates numerous databases for gene ontology and analysis, was used to conduct further pathway and process analysis of 21 proteins (14). The result showed that there were 5 pathways and processes associated with apoptosis/programmed cell death (**Figure 3C**). Six proteins (CDK5R1, ZMYND11, GNAI2, MAP2K4, PAWR, and RRM2B) were enriched in and shared by these 5 pathways and processes, of which some involved in positive regulation of apoptotic process (PAWR, MAP2K4 and CDK5R1) and some involved in negative regulation of apoptotic signaling pathway (GNAI2, ZMYND11 and RRM2B). On the other hand, the main enriched pathways and processes differed, with the top 5 were positive regulation of protein phosphorylation, growth hormone synthesis, secretion and action, response to nutrient levels, cGMP- PKG signaling pathway, and insulin resistance sorted by enriched gene counts (**Figure 3D**).

Among 21 proteins, 2 proteins (MAP2K4 and PAWR) were enriched in apoptotic processes and pathways, which indicates that they are more likely targets of miR-129-1-3p under METH treatment.

### Dual-luciferase reporter assay confirms that circ_0015891 sponges miR-129-1-3p

**Figure 4:**
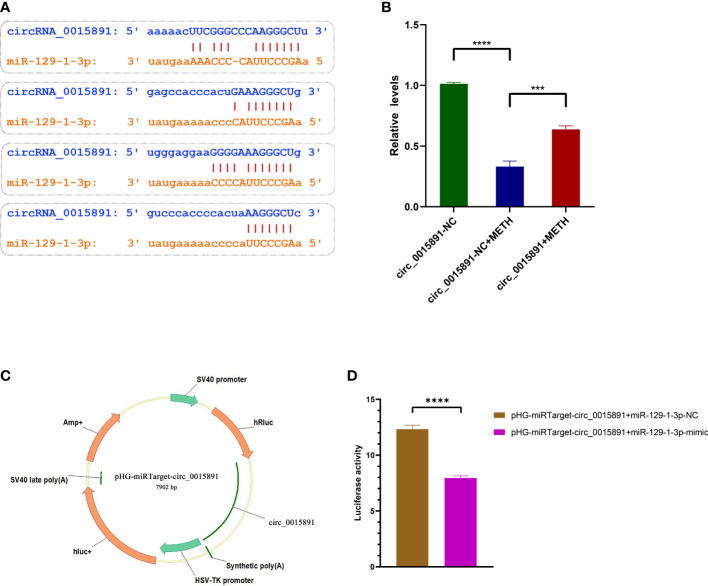
Identification and microRNA sponge validation of circ_0015891. **(A)** Base complementation prediction between circ_0015891 and miR-129-1-3p. **(B)** Relative expression levels of circ_0015891 detected by RT-qPCR. **(C)** Schematic diagram of pHG-miRTarget-circ_0015891. **(D)** Luciferase activity in groups with different co-transfection pairings. Error bars represent the mean ± SEM.***P<0.001; ****P<0.0001.

Since miR-129-1-3p inhibitor demonstrated a significant ability to repress METH-induced apoptosis, it is critical to search the upstream suppressors of miR-129-1-3. ENCORI predicted circ_0015891, one of the non-coding circRNAs transcripted by gene CSRP1, a potential sponge with 4 different types of combination of base complementation to miR-129-1-3p (**Figure 4A**) (16). RT-qPCR assays also showed a decreased expression of circ-0015891 in SH-SY5Y cells when treated with METH (**Figure 4B**). Hence, we hypothesized that circ_0015891 may regulate the apoptosis by sponging miR-129-1-3p. Dual-luciferase reporter assay was performed to validate the binding ability of circ_0015891 to miR-129-1-3p. A fluorescent reporter vector called pHG-miRTarget-circ_0015891 was conducted by inserting the sequence of circ_0015891 into the 3′ UTR of the pHG vector inserted with firefly- and Ranilla-luciferase sequences (**Figure 4C)**. The results showed that miR-129-1-3p-mimic decreased luciferase activity compared to NC when miR-129-1-3p-mimic is co-transfected with pHG-miRTarget-circ_0015891 (**Figure 4D**).

### High concentration of circ_0015891 attenuates METH-induced dopaminergic apoptosis

**Figure 5:**
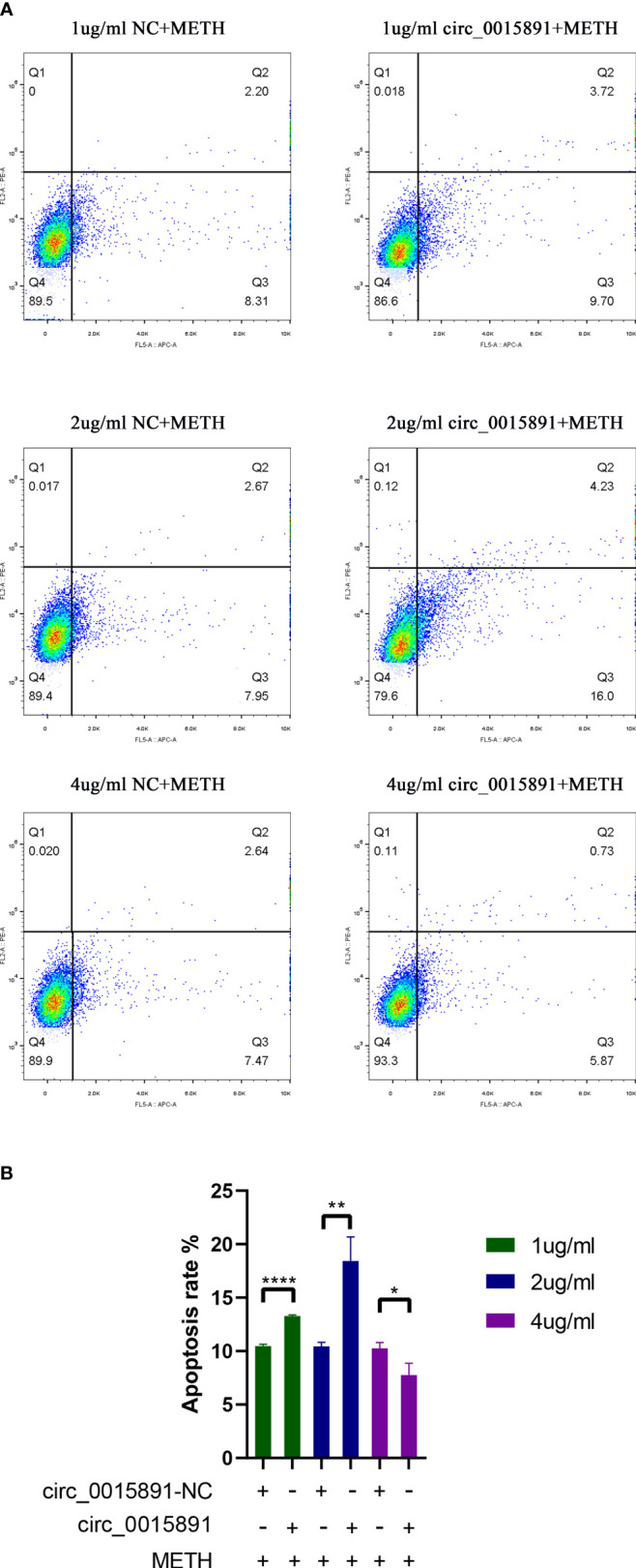
Effect of circ_0015891 in METH-induced dopaminergic apoptosis. **(A)** and **(B)** Effects of circ_0015891 on apoptosis in different concentrations. The apoptosis rate = percentage of early apoptosis (Q3) + percentage of late apoptosis (Q2). Significant differences are indicated by asterisks. Error bars represent the mean ± SEM. *P<0.05; **P<0.01; ****P<0.0001.

Effect of circ_0015891 in METH-induced apoptosis was subsequently investigated. An exogenous plasmid expressing circ_0015891 was prepared, which effectively increased the level of circ_0015891 detected by RT-qPCR assays (**Figure 4B**). Flow cytometry was conducted to detect apoptosis rate of SH-SY5Y cells treated with different concentrations of circ_0015891. The apoptosis rate was 10.46 ± 0.17% and 13.28 ± 0.10% in 1 μg/mL circRNA-NC + METH group and 1 μg/mL circ_0015891 + METH group, respectively. The apoptosis rate was 10.46 ± 0.36% and 18.43 ± 2.26% in 2 μg/ml circRNA -NC + METH group and 2 μg/ml circ_0015891 + METH group, respectively. The apoptosis rate was 10.25 ± 0.54% and 7.76 ± 1.10% in 4 μg/ml circRNA -NC + METH group and 4 μg/ml circ_0015891 + METH group, respectively (**Figure 5A**). Above all, low concentrations (1 or 2 μg/mL) of circ_0015891 promoted METH-induced apoptosis and a high concentration of 4 μg/mL of circ_0015891 exhibited the opposite effect by inhibiting METH-induced apoptosis (**Figure 5B**).

### Various miRNAs potentially sponged by circ_0015891 may influence apoptosis

**Figure 6:**
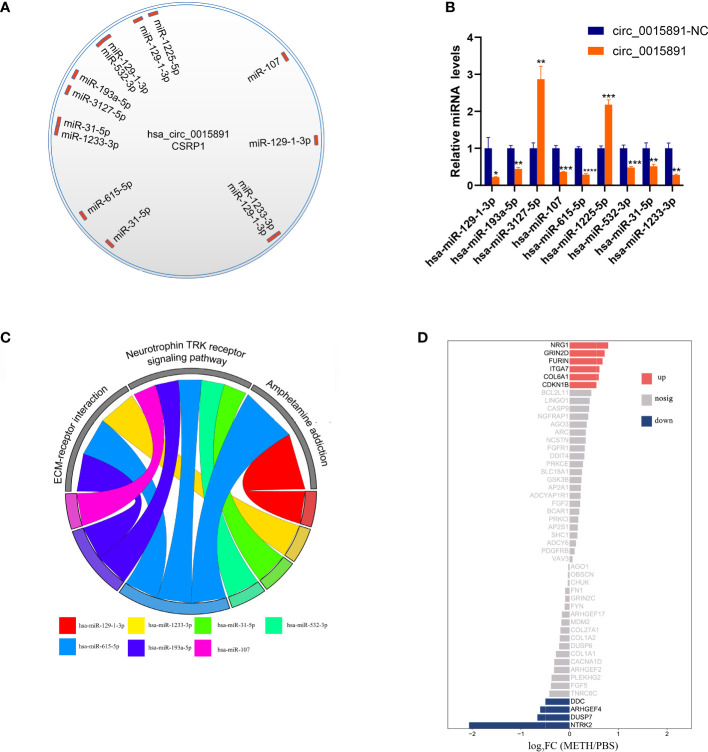
Bioinformatic analysis of circ_0015891 sponged miRNAs. **(A)** The predicted miRNAs that could bind with circ_0015891. **(B)** Relative levels of predicted miRNAs with or without circ_0015891-treatment detected by RT-qPCR assay. **(C)** String diagram of eligible miRNAs and pathways. Each color represents a miRNA. The string connecting the pathway and the miRNA represents the involvement of the miRNA in the pathway. **(D)** Bi-directional bar graph of proteins. *P < 0.05; **P < 0.01; ***P < 0.001; ****P < 0.0001.

As illustrated before, circ_0015891 exerted opposite effects on apoptosis at high versus low concentrations, which elicited our further interest. We hypothesized that circ_0015891 may bind diverse miRNAs that differ in the role to apoptosis to its different sites cause circ_0015891 is a large circRNA with a spliced sequence length of 1653 nt. ENCORI was utilized to predict miRNAs that possibly sponged by circ_0015891 and the result showed that the quantity of potentially targeted miRNAs is more than 80 (16). For reduction of interference, we screened these 80 miRNAs, leaving only those that have been clearly reported to effect neuron apoptosis in articles included in the PubMed database. Eligible miRNAs contain miR-129-1-3p, miR-1233-3p, miR-31-5p, miR-532-3p, miR-1225-5p, miR-615-5p, miR-107, miR-3127-5p and miR-193a-5p, which occupies distinct binding sites of circ_0015891 (**Figure 6A**). RT-qPCR assays showed that 7 of these miRNAs (except for miR-3127-5p and miR-1225-5p) decreased expression under circ_0015891 treatment, which indicated that they may be sponged by circ_0015891 and thus degraded (**Figure 6B**).

Further bioinformatic analysis of 7 miRNAs pathway was performed using DIANA-miRPath tool (15). Three pathways captured our attention: ECM-receptor interaction, neurotrophin TRK receptor signaling pathway and amphetamine addiction. They have at least 2 miRNAs involved and crucial relationship with pathophysiological mechanisms associated with METH (**Figure 6C**). The amphetamine addiction pathway contains 2 miRNAs that including miR-129-1-3p, which indicates this significantly up-regulated miRNA under METH treatment may possess widespread impact (**Figure 6C**). Also, the proteins that involved in these 3 pathways and predicted targets of these miRNAs were analyzed together with the transcriptome results. Under METH treatment, 6 proteins (NRG1, GRIN2D, FURIN, ITGA7, COL6A1 and CDKN1B) were significantly up-regulated and 4 proteins (DDC, ARHGEF4, DUSP7 and NTRK2) were significantly down-regulated with 39 proteins that showed no significant expression difference (**Figure 6D**). Among the 10 significantly differentially expressed proteins, 6 proteins (NTRK2, CDKN1B, ARHGEF4, DUSP7, FURIN and NRG1) are involved in neurotrophin TRK receptor signaling pathway, 2 proteins (ITGA7 and COL6A1) are involved in ECM-receptor interaction pathway, 2 proteins (DDC and GRIN2D) are involved in amphetamine addiction pathway (**Table 1**).

**Table 1:** Pathway and potential miRNA target of 10 significantly differentially expressed proteins.

Pathway	Protein	Potential miRNA target
neurotrophin TRK receptor signaling pathway	NRG1	miR-193a-5p
	CDKN1B	miR-532-3p
	ARHGEF4	miR-615-5p
	DUSP7	
	FURIN	
	NTRK2	
amphetamine addiction	DDC	
	GRIN2D	
ECM-receptor interaction	ITGA7	
	COL6A1	miR-1233-3p

## Discussion

In the present study, we identified and determined the role of miR-129-1-3p/circ_0015891 axis in dopaminergic apoptosis induced by METH and described its potential mechanisms by bioinformatic analysis. MiR-129-1-3p showed deleterious effects on dopaminergic cells under METH treatment. This effect could be blocked by the high concentration of circ_0015891, although the low concentration of circ_0015891 seemed to present the same effect as miR-129-1-3p. This opposite property of circ_0015891 reflects its complexity in bioregulation and challenges the clinical application of circ_0015891 dosing. CircRNAs with this property may be much more than circ_0015891, and any circRNA with a longer sequence resulting in an abundance of binding sites may have different effects depending on the doses.

ENCORI, the encyclopedia of RNA interactomes, predicted that 9 miRNAs related to neuron apoptosis could be sponged by circ_0015891. After performing RT-qPCR assays, 7 miRNAs which showed decreased expression under circ_0015891 treatment were further investigated (**Figures 6B, C**). With the help of the DIANA-miRPath tool, ECM-receptor interaction, neurotrophin TRK receptor signaling pathway and amphetamine addiction were considered to explain the role of these miRNAs by enriching their possibly targeting proteins.

TGA7, one of miR-615-5p’s targets, is known as a cell surface receptor for laminin-1 that mediates ECM adhesion and regulates various cellular processes (23). A study investigated that inhibition of ITGA7 down-regulated the expression of BCL2 and increased the BAX/BCL2 ratio, causing apoptosis of SH-SY5Y cells in Parkinson’s disease mouse models (24). The other ECM protein COL6A1 that targeted by miR-1233-3p, was mostly found on the close periphery of the cell surface and considered a neuroprotective role against the toxicity of amyloid-β peptides in Alzheimer’s disease mouse models (25). Given that METH can often lead to pathological changes similar to those seen in Alzheimer’s disease and Parkinson’s disease (26), the role of ITGA7 and COL6A1 in METH-induced neurotoxicity warrants deeper understanding.

As a crucial member in the neurotrophin TRK receptor signaling pathway, NTRK2 is the only receptor of brain-derived neurotrophic factor (BDNF) except for p75 neurotrophin receptor, which is thought to be a target of miR-615-5p (27) (**Table 1**). Due to the high abundance of BDNF in the brain, BDNF was the most studied among all 4 neurotrophins in the past (28). Hence the receptor NTRK2 has also received considerable attention from researchers.

Numerous studies have demonstrated that BDNF/NTRK2 axis regulates a variety of physiological processes, including dendritic branching and dendritic spine morphology (29–31), as well as synaptic plasticity and long-duration enhancement; it also plays a role in the survival, proliferation and differentiation of neural stem cells (32, 33). A research report noted that rat brains exhibited significant increases in time- and location-dependent BNDF and NTRK2 expression following binge-like METH exposure (4 x 4 mg/kg, s.c., 2 h (h) apart) (34). Another study concluded that high expression of BDNF and NTRK2 contributed to the reduction of METH-induced apoptosis (35). Combined with the 2 studies, it is reasonable to speculate that the high concentration of circ_0015891 could sponge miR-615-5p to disinhibit NTRK2 (**Table 1**), contributing to protect dopaminergic cells from apoptosis.

It is worth mentioning that not only in GO analysis from David but also in pathway/process analysis from Metascape in the downstream of miR-129-1-3p, response to nutrition was the focus of attention. MTHFR, mTOR, MAPK3, PRKCE, BMPR2, CREB1, TFRC and GNAI2 were picked up, in which TFRC, mTOR and GNAI2 were enriched by both GO and Metascape. mTOR as a hub gene in many physiological or pathological processes has been reported to have multiple roles in METH-related pathways, including apoptosis, autophagy, endoplasmic reticulum stress and oxidation (36–39). Compared to mTOR, TFRC and GNAI2 have received much less attention without any research report related to METH, which indicates a more focus needed to them.

A summary of the above discussion leads to the conclusion that abnormalities in neurotrophic receptors and responses may be an essential part of METH-induced neurotoxicity. This is reflected in downstream signaling regulated by both miR-129-1-3p and miRNAs sponged by circ_0015891.

Another point worth discussing is that amphetamine addiction pathway which enriched DDC and GRIN2D. In the classical mechanism of methamphetamine addiction, methamphetamine replaces DAT, VMAT-2, NET, and SER because of its structural similarity, followed by reversing their endogenous functions and thus redistributing monoamines from stored vesicles into the cytoplasm (40). This process leads to the release of dopamine, norepinephrine, and 5-hydroxytryptamine (5-HT) into the synapse, following which the postsynaptic monoamine receptors are stimulated (40). It has been demonstrated that monoamine receptor dopamine D2 receptor is deeply involved in single METH-induced behavioral sensitization in mice, which can be prevented by typical antipsychotic haloperidol and atypical antipsychotic risperidone, two marketed drugs that target dopamine D2 receptors (41). Proteins related to METH responses serotonin pathway is also thought to be involved in METH-induced behavioral changes. Targeting 5-HT_2C_R can reverse depressive and anxiety behaviors induced by chronic METH administration (42). Another study evaluated the role of dopamine receptors (D1R and D2R) in METH-induced presynaptic and postsynaptic damage including dopaminergic apoptosis and DA-terminal marker depletion, and found that inhibition of either of the two receptors effectively reduced presynaptic and postsynaptic damage induced by METH (43). It follows that METH-induced apoptosis of dopaminergic cells and addiction share some of the effectors and mechanisms.

Of proteins enriched in amphetamine addiction pathway, DDC is a coding protein that catalyzes the decarboxylation of DOPA to dopamine, L-5-hydroxytryptophan to 5-HT and L-tryptophan to tryptamine. In a study exploring the anti-cancer mechanism of Docetaxel and Mitoxantrone, evidence that DDC especially neural type DDC promote apoptosis was uncovered (44). Although this effect was not found in neuronal cells, but in human prostate and breast cancer cells, it suggests that DDC may have the same pro-apoptotic effect on dopaminergic cells. The other significantly DEG that enriched in amphetamine addiction pathway is GRIN2D, one of the 4 NMDAR2 (GRIN2) subunits. NMDARs are a species of ionotropic glutamate receptors controlled the opening and closing of NMDA channel which has been confirmed to be involved in long-term potentiation, an activity-dependent increase in the efficiency of synaptic transmission considered to regulate learning and memory (45). GRIN2D is a ligand-gated ion channel with high calcium permeability that causes intracellular calcium overload in pathological states contributing to induce developmental and epileptic encephalopathies (46). METH exposure and stress could change the excitability of hippocampal slices in low-magnesium epilepsy model in adult male rats with a high concentration (5mg/kg) to enhance epileptiform discharge and that a low concentration (1mg/kg) to inhibit (47). Moreover, Calcium overload mediated by GRIN2D if occurs in the heart, also causes severe cardiac cytotoxicity, such as significant myocardial apoptosis (48). Combined with the above several studies, the role of GRIN2D in METH-induced neurotoxicity including apoptosis and epileptiform lesions are worth further investigation.

**Figure 7:**
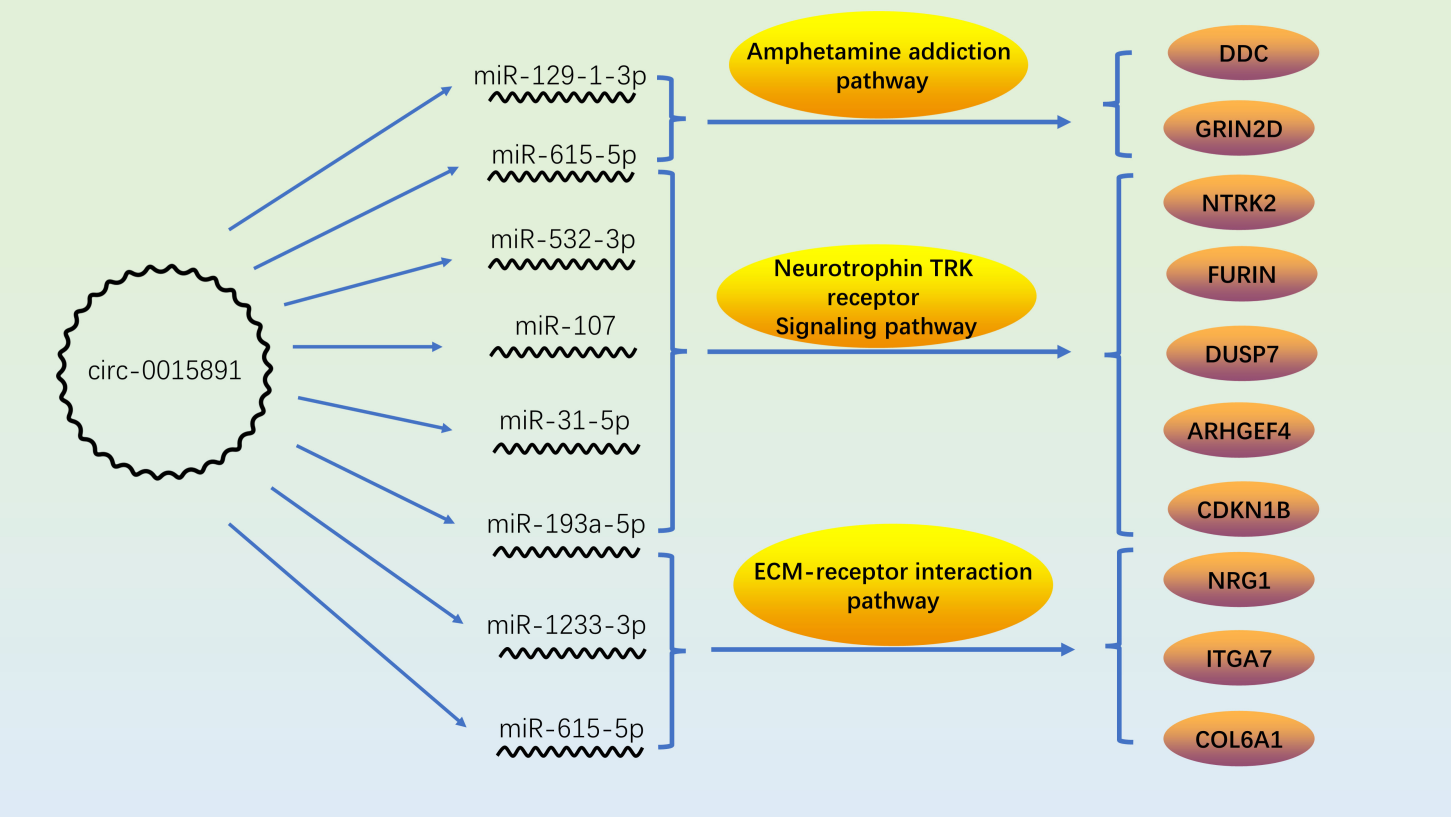
Role and correlation of circ_0015891/miRNAs/proteins in METH-related pathways.

Taken together, our data demonstrate that the circ_0015891/miR-129-1-3p axis is involved in the regulation of apoptosis of dopaminergic cells under METH treatment. Our findings complement the role of the circRNA/miRNA axis in METH-related pathways, suggest a potential downstream regulatory mechanism of miR-129 in METH-induced dopaminergic apoptosis, and provide some ideas for reference to explain the complex manifestation of circ_0015891 on apoptosis regulation (**Figure 7**).

## Data availability statement

The data presented in the study are deposited in Gene Expression Omnibus (GEO) repository, accession number GSE212346, URL: https://www.ncbi.nlm.nih.gov/geo/query/acc.cgi?acc=GSE212346.

## Author contributions

BD and YW designed the experiments. BD and XT performed the experiments. BD conducted the bioinformatic analysis and drafted the paper. YW supervised and funded the experiments and revised the paper. All authors contributed to the article and approved the submitted version.

## Funding

This study is supported by the Undergraduate Innovation and Entrepreneurship Training Program Supportive Project (grant no. 2020105330143).

## Conflict of interest

The authors declare that the research was conducted in the absence of any commercial or financial relationships that could be construed as a potential conflict of interest.

## Publisher’s note

All claims expressed in this article are solely those of the authors and do not necessarily represent those of their affiliated organizations, or those of the publisher, the editors and the reviewers. Any product that may be evaluated in this article, or claim that may be made by its manufacturer, is not guaranteed or endorsed by the publisher.

## Abbreviations

BBC3, BCL2 binding component 3; MAP2K4, mitogen-activated protein kinase kinase 4;PRKCE: protein kinase C epsilon; PAWR, pro-apoptotic WT1 regulator; MAPK3, mitogen-activated protein kinase 3; CDK5R1, cyclin dependent kinase 5 regulatory subunit 1; ZMYND11, zinc finger MYND-type containing 11; GNAI2, G protein subunit alpha i2; RRM2B, ribonucleotide reductase regulatory TP53 inducible subunit m2b; BCL-2, B-cell CLL/lymphoma 2; DAT, dopamine transporter protein; NET, norepinephrine transporter protein; SERT, serotonin (5-HT) transporter protein; VMAT-2, vesicular monoamine transporter protein-2; DUSP-7, dual specificity phosphatase 7; DDC, dopa decarboxylase; GRIN2D, glutamate ionotropic receptor NMDA type subunit 2d; NMDAR2, N-methyl-D-aspartate receptor 2; NRG1, neuregulin 1; FURIN, paired basic amino acid cleaving enzyme; ITGA7, integrin subunit alpha 7; COL6A1, collagen type VI alpha 1 chain; CDKN1B, cyclin dependent kinase inhibitor 1b; ARHGEF4, rho guanine nucleotide exchange factor 4; NTRK2, neurotrophic receptor tyrosine kinase 2; 5-HT_2C_R, 5-HT2C receptor.
