# Supplementary material for: Regulation and bioinformatic analysis of circ_0015891/miR-129-1-3p axis in methamphetamine-induced dopaminergic apoptosis
Source: Front Endocrinol (Lausanne). 2022 Sep 20;13:999211. doi: 10.3389/fendo.2022.999211 (PMC9530452; doi:10.3389/fendo.2022.999211)
Supplement: Supplementary file 3 [file DataSheet_3.zip › Raw data type in Excel/binging sites of mir-129 and circ_0015891.docx]

**circRNA_0015891: 5' aaaaacUUCGGGCCCAAGGGCUu 3'**
                   
**miR-129-1-3p: 3' uaugaaAAACCC-CAUUCCCGAa 5**

**|| |||   |||||||**

**circRNA_0015891: 5' gagccacccacuGAAAGGGCUg 3'**
                         
**miR-129-1-3p: 3' uaugaaaaacccCAUUCCCGAa 5'**

**| |||||||**

**circRNA_0015891: 5' ugggaggaaGGGGAAAGGGCUg 3'**
**|||| |||||||**
**miR-129-1-3p: 3' uaugaaaaaCCCCAUUCCCGAa 5'**

**circRNA_0015891: 5' gucccaccccacuaAAGGGCUc 3'**
**|||||||**
**miR-129-1-3p: 3' uaugaaaaaccccaUUCCCGAa 5'**
